# Inhibition of Aβ_42_ oligomerization in yeast by a PICALM ortholog and certain FDA approved drugs 

**DOI:** 10.15698/mic2016.02.476

**Published:** 2016-01-20

**Authors:** Sei-Kyoung Park, Kiira Ratia, Mariam Ba, Maria Valencik, Susan W. Liebman

**Affiliations:** 1 Present address: Department of Biochemistry and Molecular Biology, University of Nevada, Reno, Reno, NV, USA.; 2 HTS facility, Research Resources Center, University of Illinois, Chicago, Chicago, IL 60612, USA.; 3 Department of Biological Sciences, University of Illinois, Chicago, Chicago, IL 60607, USA.

**Keywords:** Aβ_42_ oligomerization, yeast, HTS, PICALM, Alzheimer

## Abstract

The formation of small Aβ_42_ oligomers has been implicated as a toxic species in Alzheimer disease (AD). In strong support of this hypothesis we found that overexpression of Yap1802, the yeast ortholog of the human AD risk factor, phosphatidylinositol binding clathrin assembly protein (PICALM), reduced oligomerization of Aβ_42_ fused to a reporter in yeast. Thus we used the Aβ_42_-reporter system to identify drugs that could be developed into therapies that prevent or arrest AD. From a screen of 1,200 FDA approved drugs and drug-like small compounds we identified 7 drugs that reduce Aβ_42_ oligomerization in yeast: 3 antipsychotics (bromperidol, haloperidol and azaperone), 2 anesthetics (pramoxine HCl and dyclonine HCl), tamoxifen citrate, and minocycline HCl. Also, all 7 drugs caused Aβ_42_ to be less toxic to PC12 cells and to relieve toxicity of another yeast AD model in which Aβ_42_ aggregates targeted to the secretory pathway are toxic. Our results identify drugs that inhibit Aβ_42_ oligomers from forming in yeast. It remains to be determined if these drugs inhibit Aβ_42_ oligomerization in mammals and could be developed as a therapeutic treatment for AD.

## INTRODUCTION

Alzheimer’s disease (AD), a progressive and fatal brain disorder, is the most common form of dementia currently affecting more than 5 million Americans. Furthermore, more than 26 million people worldwide have some form of dementia.

With the increase in the age of the population, the number of AD patients is expected to triple by 2050 causing a staggering emotional and financial toll. Unfortunately, there is no prevention or satisfactory treatment to date.

To prevent or stop disease progression at an early stage, we need to attack the underlying causes of the disease. The major physical feature of AD is the accumulation of abnormally folded beta-amyloid (Aβ) and Tau in the brain. When Aβ_42 _is polymerized *in vitro* 1,2,3,4 or purified from *in vivo* animal models, post mortem brain 5,6 or cerebrospinal fluid (CSF) tissue of AD patients 7,8, small Aβ_42_ aggregates are found that differ in size and shape. Such small aggregates of the Aβ_42_ peptide (dimers, trimers, tetramers, etc.) appear to be neurotoxic because they trigger abnormalities in neuronal excitation and synaptic plasticity, and inhibit hippocampal long-term potentiation 1,2,9,10,11,12. Still, it remains to be determined if Aβ_42 _is a major cause of AD 13. Nonetheless, there is evidence for a pathological role of Aβ oligomers on other protein oligomers in neurodegenerative conditions, such as Parkinson's disease 14, and prion diseases 15.

Many fundamental biological processes and pathways such as chaperone and protein remodeling, the ubiquitin proteasome system, secretion, vesicular trafficking, and autophagy, are highly conserved between yeast and human cells. Indeed, yeast models have become powerful tools for unraveling the molecular basis of complex human neurodegenerative diseases 16,17,18,19. Treatment with Aβ_42_ oligomers formed *in vitro* or expression of Aβ_42_ oligomers *in vivo* affects the growth of yeast cells 20,21,22,23,24. Also, a yeast model in which Aβ_42_ (referred as HDEL-Aβ_42_) is toxic was recently developed. Here, a *GAL1 *promoter was used to express a *KAR2* signal sequence (*HDEL*) Aβ_42_ fusion protein. This Aβ_42_ fusion protein was directed to the secretory pathway where it disrupted normal cellular endocytic trafficking, causing toxicity 24. Importantly, overexpression of *YAP1802*, a yeast homolog of PICALM, rescued cells from this toxicity 24. PICALM (phosphatidylinositol clathrin assembly lymphoid-myeloid leukemia) protein plays a key role in a clathrin-mediated endocytosis and genome-wide association studies identified single nucleotide polymorphisms in the gene of PICALM as genetic risk factors for late-onset AD 25,26. Furthermore, overexpressed PICALM protected primary rat cortical neurons and *C. elegans* from toxicity of extracellular aggregated Aβ_42_ oligomers. In addition, PICALM affected Aβ_42 _toxicity in a yeast model in which the α-factor signal sequence was fused to Aβ_42_, although here overexpression of PICALM enhanced toxicity 23. While little is known about the contribution of PICALM to AD pathogenesis, these findings strongly support the hypothesis that Aβ_42_ is associated with AD toxicity.

Yeast has been used to screen for chemical compounds that reduce aggregation or oligomerization of the Aβ peptide by assaying for the activity of reporters fused to Aβ 27,28. Previously, using a yeast Aβ_42 _oligomerization model in which Aβ_42_ was fused to the functional release factor (RF) domain of yeast translational termination factor, Sup35 29, we screened for anti-Aβ_42_ oligomer compounds 28. This Aβ_42_-RF fusion formed SDS-resistant low-n oligomers that reduced release factor activity, thus enhancing a read-through of stop codon mutations. Indeed, a correlation of oligomer formation and stop codon read-through was confirmed by biochemical analysis 28,29. An important distinction of this approach from previous anti-Aβ aggregation screens 30,31,32 is that we can detect drugs that inhibit Aβ_42_ oligomer formation but do not inhibit the formation of large Aβ_42 _amyloid. This is important because such large aggregates are now thought to be helpful because they likely capture some of the more toxic Aβ_42_ oligomers, rendering them less toxic 33,34

Here, we show that the mechanism of the PICALM, human AD risk factor, is likely to reduce the level of Aβ_42_ oligomers in cells. This strongly supports the hypothesis that oligomerization of Aβ_42 _is a major cause of AD toxicity. We then screened FDA-approved drugs that could readily be developed into Alzheimer’s therapies, to identify drugs that prevent the formation of Aβ_42_ small oligomers using the yeast Aβ_42_-RF reporter system. We also showed that each of the drug hits counteract yeast and mammalian cell toxicity associated with Aβ_42_ small aggregates.

## RESULTS

### YAP1802, homolog of PICALM, inhibits Aβ_42_-RF oligomer formation 

**Figure 1:**
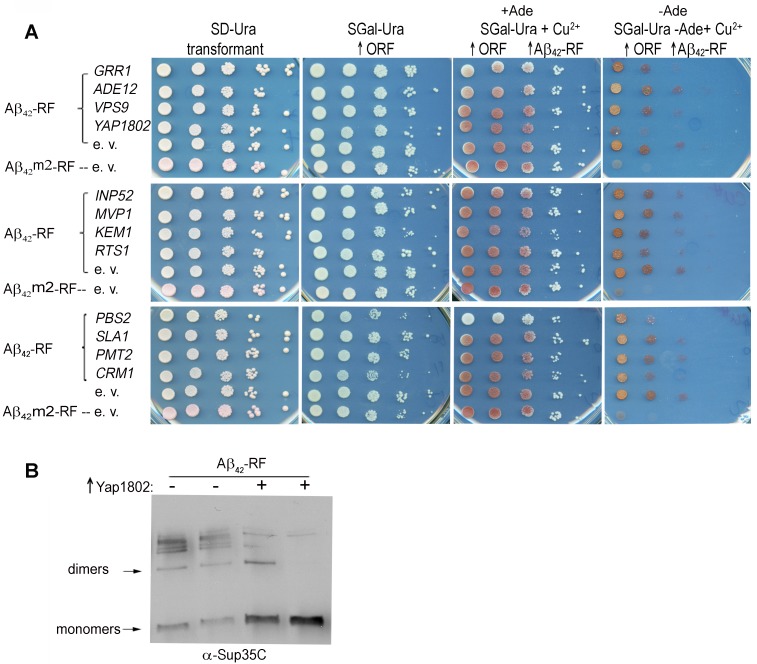
FIGURE 1: Effect of genetic modifiers of toxic HDEL-Aβ_42_ on Aβ_42_-RF oligomer formation and Aβ_42_-MRF aggregates into toxic SDS-stable oligomers in yeast. **(A)** Overexpressed Yap1802 but not other previously described 24 HDEL-Aβ_42_ modifiers dramatically enhanced the translational termination factor activity of Aβ_42_-RF. Aβ_42_-RF expressing cells (*CUP1::Aβ_42_-RF*) lacking chromosomal *SUP35* were transformed with a plasmid carrying the indicated gene under control of the *GAL1* promoter. Ten-fold serial dilutions of transformants are shown on plasmid selective non-inducing media (SD-Ura); galactose plasmid selective media (SGal-Ura) to turn on each gene (↑ORF); +Ade medium containing copper to overexpress Aβ_42_-RF (↑ORF ↑Aβ_42_-RF); the identical -Ade medium SGal-Ura-Ade+Cu^2+^ (-Ade ↑ORF ↑Aβ_42_-RF) to determine translational termination factor activity as read-through of the *ade1-14 *nonsense mutant. The Aβ_42_-RF overexpressed in cells was shown to have reduced translational termination factor activity as cells grew on -Ade due to aggregation of the fusion protein into small oligomers (Aβ_42_-RF-e. v.). However, the translation termination factor activity was retained in yeast cells overexpressing Aβ_42_m2-RF (Aβ_42_ aggregation-deficient mutant) or YAP1802*, *due to the absence of oligomer formation, resulting in reduced growth on -Ade (Aβ_42_m2-RF-e. v.). **(B)** Yap1802 suppression of Aβ_42_-RF oligomerization by immunoblot analysis. Total cell lysates were prepared from 2 independent transformants of an Aβ_42_-RF strain carrying *YAP1802* (+) or an empty vector (-) plasmid. Both Aβ_42_-RF and YAP1802 were overexpressed (SGal-Ura-Ade+Cu^2+^) prior to lysis. Immunoblots were probed with anti-Sup35 RF to evaluate the level of oligomers and monomers. ↑Yap1802 indicates overexpression of *YAP1802*. The identification of bands as Aβ_42_-RF dimers and monomers was determined by the estimated sizes of the bands in the immunoblot.

We tested whether genetic modifiers that rescue HDEL-Aβ_42_ toxicity 24 likewise repair the compromised Aβ_42_-RF translational termination factor activity due to reduced Aβ_42_-RF oligomer formation using the above described growth assay 28,29. The cell growth phenotype in this assay requires expression of full length Ade1. The impaired Sup35 translational release factor (RF) activity of oligomerized Aβ_42_-RF allows read-through of the *ade1-14* premature stop codon enabling growth on adenineless media (-Ade) (Aβ_42_-RF-empty vector (e. v.) in Figure 1A). However in the presence of drugs or genetic modifiers blocking oligomerization of Aβ_42_-RF, or in cells expressing a fusion made with the Aβ_42_ aggregation-deficient mutation 28,29, Aβ_42_m2-RF, the RF activity is restored, so cells cannot grow on -Ade (Aβ_42_m2-RF-empty vector (e. v.) in Figure 1A). Among the 12 genetic modifiers identified in the HDEL-Aβ_42_ screen 24, only cells expressing *YAP1802* regained translational termination factor activity (shown as reduced growth on -Ade), suggesting it restored Aβ_42_-RF to the soluble monomeric state (Figure 1A). Indeed, immunoblots developed with Sup35C antibody showed that the level of SDS-resistant Aβ_42_-RF oligomer was significantly reduced, and the level of the monomers was significantly increased when Yap1802 was overexpressed (Figure 1B). Thus, *YAP1802* is likely to affect toxicity by reducing the level of toxic Aβ_42 _oligomers.

We also used NAB61 antibody, which was reported to preferentially recognize toxic Aβ oligomers in AD patients 35 and HDEL-Aβ_42_ in yeast 24. Indeed, while Sup35C antibody detected only monomers in Aβ_42_m2-RF cells expressing the Aβ_42_ aggregation-deficient mutation (Figure S1), NAB61 did not recognize Aβ_42_m2-RF monomers although it detected a species the size of dimers. However, in our hands NAB61 recognized both oligomers and monomers of wild-type Aβ_42_-RF (Figure S1).

### Screen for drugs that inhibit Aβ_42_-RF from forming toxic oligomers 

We screened the Prestwick Chemical Library® (Prestwick Chemical, Washington, DC) which contains 1,200 FDA approved drugs and drug-like molecules for the ability to reduce Aβ_42_ oligomer formation using the growth assay described above 28,29. Drugs that blocked oligomerization of Aβ_42_-RF, thereby restoring the RF activity and preventing stop codon read-through, were selected as initial candidates because they reduced growth in -Ade.

**Figure 2:**
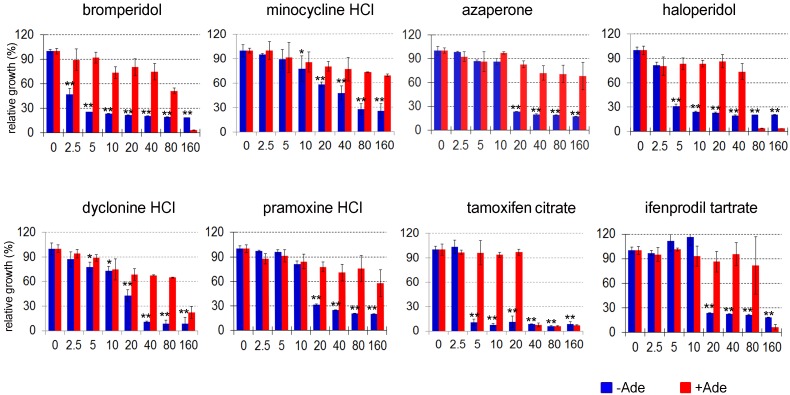
FIGURE 2: Dose-dependent effects of 8 drugs on Aβ_42_-RF activity as measured by cell growth. Aβ_42_-RF expressing cells were treated with each drug at the indicated concentrations. Their effects on cell growth were measured as OD_600_ in +Ade and -Ade media after 4 and 5 days respectively (see Materials and Methods for details). Growth in -Ade is dependent upon Aβ_42_-RF release factor activity. This activity is expected to be reduced by oligomerizartion. Growth on +Ade does not require Aβ_42_-RF release factor activity and is used as a control in the presence of each drug. Shown is the relative growth in the presence vs. absence of each drug. Error bars show the standard deviation from three replicates. The asterisks show significance levels of **P < 0.01 or *P < 0.05 according to the Student’s t test between DMSO (0) control and drug treatment at marked concentration for growth in -Ade.

Each assay was carried out in duplicate at a single compound concentration (20 µM). The Z values 36 calculated from controls were 0.65 and 0.71 for the -Ade and +Ade assays, respectively, indicating the assay qualities were excellent. In the initial screen, 24 known drugs from among the 1,200 compounds in the library emerged as candidates that inhibited growth in -Ade medium without significant inhibition of growth in +Ade medium. When the results were repeated, 14 of the 24 drugs at 20 µM in DMSO inhibited growth more than 50% in -Ade when compared to a no drug DMSO control (the 8 drugs in Figure 2 and the 6 drugs written in bold in Figure S2). The 6 hits shown in Figure S2 were dropped because they also decreased growth in +Ade medium by ≥30% compared to the no drug control, indicating they are generally toxic to yeast, which invalidates our -Ade growth assay.

### Dose-dependent inhibition of Aβ_42_-RF oligomerization

The 8 drugs that passed the confirmation screen were tested for dose-dependent effects on Aβ_42_-RF activity. The percent of relative growth in the presence of the drugs dissolved in DMSO was determined from the OD_600_ (Figure 2). The Aβ_42_-RF control treated with DMSO only, showed good growth in -Ade, indicating Aβ-RF associated translational termination activity is compromised in the absence of drug due to oligomerization of the fusion protein. Aβ_42_m2-RF with DMSO had little or no growth in -Ade, indicating the mutant fusion protein was functional and not aggregated (Figures 1A and S1). In the presence of each drug, Aβ_42_-RF showed dose-dependent growth inhibition in -Ade, but no or mild growth inhibition in +Ade, indicating that the drugs block Aβ_42_-RF activity likely by blocking oligomerization.

**Figure 3:**
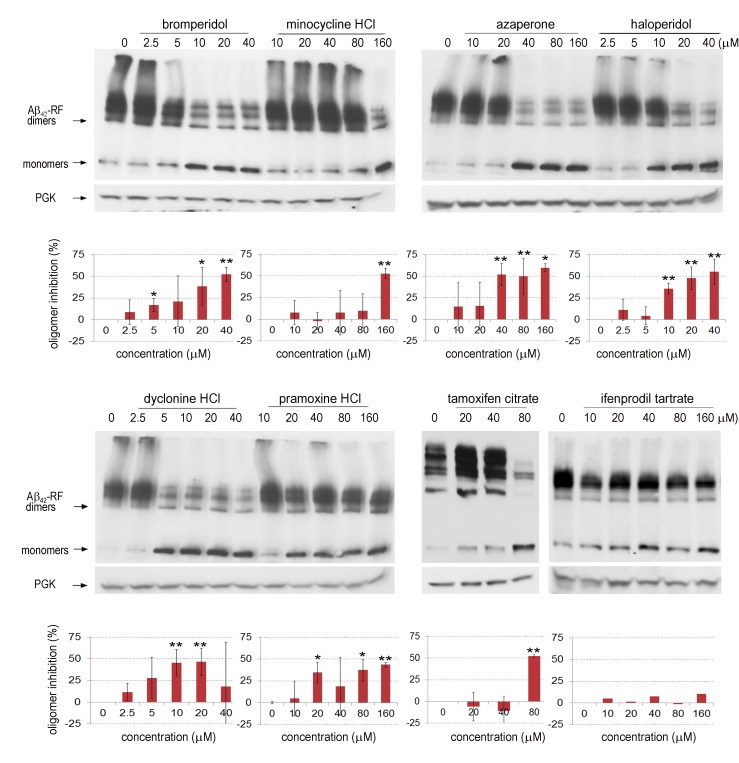
FIGURE 3: Seven drugs suppress Aβ_42_-RF oligomerization in yeast in a dose-dependent manner. SDS-resistant Aβ_42_-RF oligomers were detected by immunoblot analysis (upper). The assay strain expressing Aβ_42_-RF was grown in complex medium in the presence of each compound at the indicated concentrations. Equal amounts of lysate proteins were treated with 1% SDS for 7 mins at room temperature and analyzed by SDS PAGE followed by immunoblotting with anti-Sup35 RF antibodies. Aβ_42_-RF cells grown in DMSO (0) were used as controls. PGK, yeast 3-Phosphoglycerate Kinase, detected with anti PGK antibodies was an internal control to show the effect of the drugs on protein synthesis in general. Immunoblot signals for Aβ_42_-RF monomers and oligomers were quantified and converted into % inhibition of oligomer formation from the ratios of oligomers to monomers compared to DMSO controls (0 means no inhibition) (lower). Error bars represent standard deviation from 3 independent immunoblots except for ifenprodil tartrate which shows data from 1 of 2 immunoblots that showed no drug effects. The asterisks show significant levels of **P < 0.01 or *P < 0.05 according to Student’s t test.

We analyzed lysates prepared from cells grown with various concentrations (0-40 or 0-160 µM) of each drug to test whether the restored translational termination factor activity associated with the drugs is correlated with a decrease in the level of SDS-resistant Aβ_42_-RF oligomers. In cells grown with DMSO only (the first lane marked 0 in each gel in immunoblots in Figure 3), there were mostly SDS-resistant Aβ_42_-RF oligomers (dimers, trimers and tetramers, etc.) and few monomers. Higher drug concentrations decreased the levels of oligomers and concomitantly increased the level of monomers, while the drugs have no effect on 3-phosphoglycerate kinase (PGK) expression at any concentration. This inhibition of oligomer formation was quantified by comparing the ratios of oligomers to monomers detected on immunoblots of cell lysates grown in the presence or absence of drug (Figure 3 shows a representative sample from 3 independent experiments). Bromperidol, azaperone, haloperidol, pramoxine HCl, and dyclonine HCl exhibited strong anti-oligomer activity (Figure 3) as well as restored Aβ_42_-RF activity indicated by growth inhibition in -Ade at relatively low concentrations (Figure 2). Unexpectedly, minocycline HCl and tamoxifen citrate, that decreased growth in -Ade at 10-20 µM and 5 µM, respectively (Figure 2), only showed inhibition of oligomer formation at high concentrations, 160 and 80 µM, respectively. Finally, ifenprodil tartrate that showed dose-dependent growth inhibition in -Ade failed to change the oligomer profile (Figure 3) and was dropped.

Importantly, the remaining 7 drugs enhanced the ability of Aβ_42_-RF, but not of a *SUP35 *suppressor mutation (*G1256A* 37), to terminate protein synthesis at the *ade1-14* nonsense mutation, as measured by growth on -Ade (Figure S3). Thus the drugs do not have a general antisuppressor activity, but instead specifically affect Aβ_42_-RF.

### Drugs prevent Aβ_42_ from being toxic in PC12 cells 

**Figure 4:**
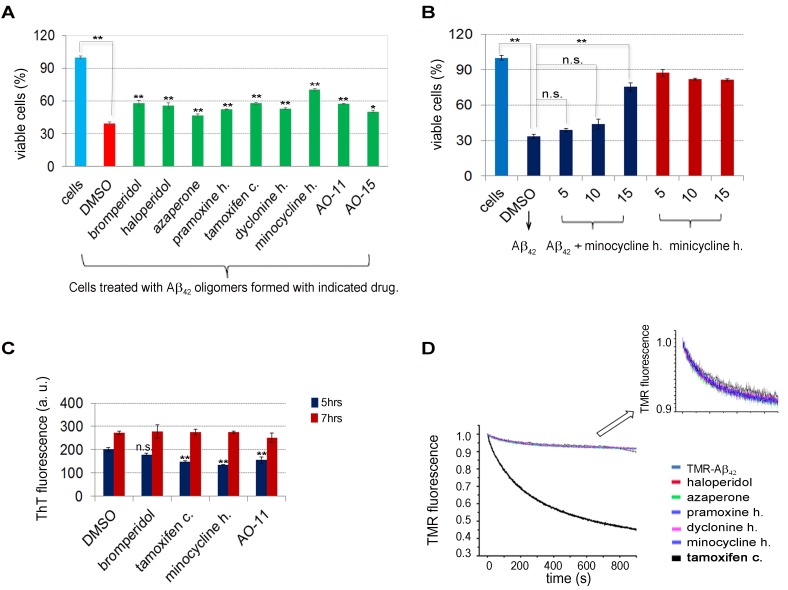
FIGURE 4: The protective effect of drugs on Aβ_42_ induced cytotoxicity in PC12 cells. **(A)** Toxicity of Aβ_42_ assembled under conditions favoring oligomer formation was reduced when assembly was done in the presence of drugs. Aβ_42_ at 200 μM assembled under conditions favoring oligomer formation in the presence of 100 μM drug or DMSO was diluted and added to PC12 cells at a concentration of 20 μM Aβ_42_ and 10 μM drug. Cell viability was assessed after 24 hrs growth at 37°C using the MTT assay. The percentage of viable cells is shown. Error bars are the standard deviation of triplicate experiments. **(B)** Dose-dependent effects of minocycline HCl on Aβ_42_ associated cytotoxicity. Methods were as in (A). PC12 cells were treated with final concentrations of 20 μM Aβ_42_ with 5, 10, and 15 µM minocycline HCl or DMSO only (blue bars). Cells were also treated with the indicated amount of minocycline HCl alone (red bars). **(C) **Effect of drugs on *in vitro* Aβ_42_ high molecular fibril assembly in fibril favorable conditions. Aβ_42_ was assembled into high molecular fibrils at 37°C in the presence of 50 µM drug or control DMSO according to 42. Thioflavine T (ThT) was added to aliquots at the indicated times and fluorescence, indicative of fiber formation, was measured. **(D) **Effect of drugs on *in vitro* labeled Aβ_42_ (TMR-K-Aβ_42_) oligomerization. Reduced TMR fluorescence, which measures oligomerization, was recorded as a function of time immediately after dilution of the TMR-K-Aβ_42_ with addition of 10 µM of each drug or DMSO for a negative control. The asterisks show significance levels of **P < 0.01 or *P < 0.05 according to Student’s t test between DMSO control and drug treatment (A) and DMSO control and drug treatment at 5 hrs (C).

Some species of Aβ oligomers of various sizes and shapes prepared *in vitro* or purified from post mortem brains have been shown to be toxic when applied to neuronal cell culture or primary cortical neurons 38,39,40,41. To test the activity of drugs in reducing Aβ_42_ oligomerization, we added the drugs as we assembled Aβ_42_ using *in vitro* conditions in which the aggregation of Aβ_42_ into small soluble oligomers was favored 38. Treatment of rat PC12 neuronal cells with 20 µM of Aβ_42_ assembled in the presence of DMSO decreased cell viability to about 40% (red bar in Figure 4A) compared to control cells treated with DMSO in the absence of Aβ_42_ (blue bar in Figure 4A), indicating that the assembled Aβ_42 _were indeed toxic. Aβ_42_ assembled in the presence of the remaining 7 hits from the Prestwick Chemical Library® were less toxic (Figure 4A), suggesting that the drugs impede the formation of the toxic Aβ_42_ assemblies. In addition, AO-11 and AO15, compounds that inhibit Aβ_42_-RF from forming oligomers, identified from our previous screen 28, also relieved toxicity.

Among drugs tested, minocycline HCl was the most effective. Cell viability increased from 40% when Aβ_42_ was assembled in DMSO alone, to 68% when it was assembled in the presence of 10 µM minocycline HCl (Figure 4A). The minocycline HCl protection was dose-dependent (Figure 4B). Furthermore, treatment of PC12 cells with minocycline HCl without Aβ_42_ did not improve cell viability (Figure 4B).This suggests the protective effect of the minocycline HCl was not due to the enhancement of cell growth but rather was due to inhibition of Aβ_42_ toxicity.

### Drugs do not affect *in vitro* Aβ_42_ fibril or oligomer formation

To test whether the drugs can also inhibit high molecular Aβ_42_ fibril formation, 4 of the most active drugs (bromperidol, tamoxifen citrate, minocycline HCl, and AO-11) were added to 20 µM Aβ_42_ at 50 µM, under conditions that promote fibrilization 42. Fibrils were allowed to form for 5 or 72 hrs and the Thioflavine T (ThT) fluorescence assay was used to quantify fibril formation. At 5 hrs there was a mild inhibition of Aβ_42 _fibrilization, but by 72 hrs there was no significant change in fibrilization relative to the DMSO control (Figure 4C). Thus, while these drugs may have some effect on initial Aβ_42_ small seed formation, they do not block fibril formation.

We also tried to test the effects of bromperidol, azaperone, pramoxine HCl, dyclonine HCl, minocycline HCl and tamoxifen citrate when added to Aβ_42_ during *in vitro* oligomerization conditions 38. However, when these reactions were run on Western blots, only Aβ_42_ the size of 12mers and larger were detected. Aβ_42 _monomers and small oligomers were not seen and the drugs had no effect on this.

### Tamoxifen citrate prevents early steps of Aβ_42_ oligomerization *in vitro*

We used an *in vitro* labeled Aβ_42_ oligomerization assay to test the effects of the drugs on the early steps of monomer to oligomer conversion 43. This assay takes advantage of the fluorescence self-quenching observed when tetramethylrhodamine (TMR) is covalently attached to the N-terminal lysine of Aβ_42_ (TMR-K-Aβ_42_) 43. The loss of TMR fluorescence indicates self-association of Aβ_42_ monomers into oligomers (e.g. dimers, trimers, etc.). Most of the drugs did not affect the formation of the early oligomers in this assay. They showed the same fluorescence changes as the control without drug (TMR-K-Aβ_42_) (Figure 4D). In contrast, tamoxifen citrate strongly reduced TMR fluorescence, indicating the formation of early off-pathway oligomers 43 that could be functional or non-functional oligomers.

### Anti-Aβ_42_-RF oligomer drugs reduce toxicity of HDEL-Aβ_42_ in yeast 

We next tested the effects of the 7 drugs we identified that impede Aβ_42_-RF oligomerization (Figures 2 and 3) on HDEL-Aβ_42_ toxicity. To do this we used a strain with a deletion of *ERG6 *(L3340), which is critical for increased drug permeability. L3340 was transformed with a 2µ plasmid (pAG425 *GAL1::HDEL-Aβ_42_*) carrying HDEL-Aβ_42_ under the *GAL1* promoter. As previously reported for *ERG6* wild-type cells 24, we found that *erg6*∆ cells expressing HDEL-Aβ_42_ were growth inhibited (middle in Figure S4) compared to controls transformed with an empty vector (top in Figure S4) and that the toxicity was suppressed by overexpressing *YAP1802* (bottom in Figure S4).

**Figure 5:**
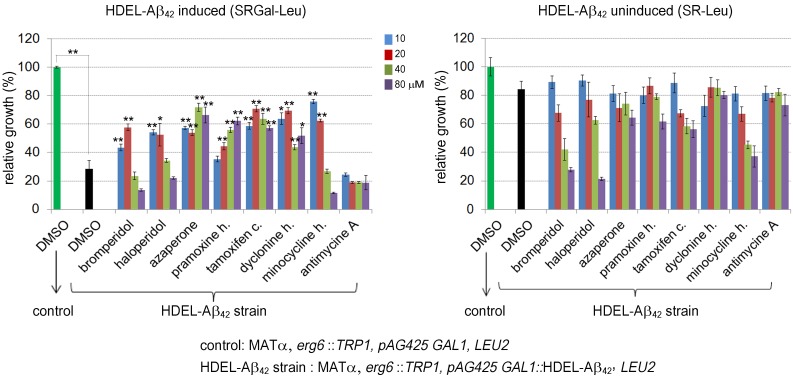
FIGURE 5: The effect of drugs that inhibit Aβ_42_-RF from forming oligomers on toxicity caused by overexpression of HDEL-Aβ_42 _in yeast. Effects of 7 drugs on suppression of HDEL-Aβ_42_ toxicity and general cell growth are shown. Yeast cells carrying either HDEL-Aβ_42_ (HDEL-Aβ_42_ strain) or an empty vector (control) grown overnight in non-inducing plasmid selective media (2% raffinose, SR-Leu) were normalized, diluted to 1 x 10^5^ cells/100 µl of media in each of 96 wells and further grown for 3 days in 2% galactose inducing media (SRGAL-Leu) or 2% raffinose noninducing media (SR-Leu) in the presence of each drug at the indicated concentrations. Growth was measured as OD_600_. Error bars show the standard deviation from three trials. The asterisks show increased relative growth with drug treatment compared to DMSO control (black bar in left panel) at significance levels of **P < 0.01 or *P < 0.05 according to Student’s t test.

We measured growth in liquid to assay the effects of drugs that inhibit Aβ_42_-RF oligomerization on the toxicity of this strain (Figure 5). Drugs at the indicated concentrations were added to yeast with the toxic HDEL-Aβ_42_ construct or an empty vector control. Cells were diluted in media that did (SGAL-Leu) or did not (SR-Leu) induce expression of HDEL-Aβ_42_. While controls carrying the empty vector grew well in both inducing and non-inducing media (dark green bars in Figure 5), growth of yeast induced to express the HDEL-Aβ_42_ (black bar in Figure 5 left) was decreased by 65% compared to growth in non-inducing media (black bar in right), verifying that HDEL-Aβ_42_ caused cytotoxicity. Treatment with 10 and 20 µM bromperidol, haloperidol and minocycline HCl relieved the toxicity while higher drug concentrations were toxic. Treatment with azaperone, pramoxine HCl, tamoxifen citrate and dyclonine HCl showed a relatively strong effect of suppressing the toxicity HDEL-Aβ_42_ (left panel Figure 5) while not having a significant effect on growth in the absence of HDEL-Aβ_42_ (right panel Figure 5). However, we were unable to detect effects of drugs tested (azaperone, pramoxine HCl, dyclonine HCl and tamoxifen citrate) on the HDEL-Aβ_42_ oligomerization. Possibly this is because oligomerization of HDEL-Aβ_42 _was very different from the oligomerization of Aβ_42_-RF, showing only HDEL-Aβ_42 _monomers and trimers.

Antimycine A, a control that showed no effect on Aβ_42_-RF oligomerization (Figure S5) was also unable to relieve toxicity of HDEL-Aβ_42_. In addition, the other 15 drugs that initially passed the Aβ_42_-RF growth inhibition screen, but that were later eliminated as candidates, all failed to rescue HDEL-Aβ_42._

## DISCUSSION 

Despite tremendous efforts, there are only a few drugs that mask or relieve the symptoms of AD. Furthermore, we do not have any medications that prevent, arrest or cure the disease (www.alz.org). Recent improved assays provide evidence for Aβ small oligomeric species in AD brains 7,8,44. While the cause of AD remains unknown, increasing evidence suggests that aggregation of the Aβ_42_ peptide into small oligomers plays an important role in disease progression 12,15,45. This hypothesis is also supported by a yeast model 24 in which toxicity associated with expression of Aβ_42 _was suppressed by overexpression of YAP1802, the yeast homolog of PICALM, a previously known genetic AD risk factor 25,46. Furthermore, in this paper we show that overexpressing Yap1802 inhibits Aβ_42_-RF aggregation into oligomeric species (Figure 1B). This provides strong support for the idea that inhibiting the initial production or aggregation of Aβ_42 _would be an effective treatment to prevent or slow disease onset.

One approach has been to prevent the production of Aβ peptide by inhibiting the secretases that cleave Aβ from the amyloid precursor protein 47,48. However, results of recent clinical trials of inhibitors of β- (LY2886721; Eli Lilly and Company, 2013 press release) and γ-secretases (Semagacestat; Eli Lilly and Company, 2010 press release and Avagacestat; Bristol-Myers Squibb, 2012 press release) have been disappointing. This makes the approach of inhibiting the formation of toxic Aβ oligomers of more interest.

Among a handful of drugs that appear to inhibit Aβ_42_ from forming toxic oligomers 6,27,32,41,49,50,51,52,53 in pilot studies, several have reached clinical trials. Indeed, trials of PBT1 (clioquinol), a metal chelator, which modulates affinity for Cu^2+^ and Zn^2+^ and inhibits metal-induced Aβ_42_ aggregation 6,54,55 were dropped because of side effects 56. PBT2, a second generation of PBT1, was safe but demonstrated no significant effect on cognition or memory in the trial (Pubmed Health, 2014). Phase 2 trials of scyllo-inositol (ELND005), that targets the C-terminus of Aβ_42_ and neutralizes cell derived Aβ_42_ trimers 57, were completed with promising results (Transition Therapeutics, Inc., 2014 news press).

Finding good drug candidates through high-throughput screening (HTS) is a long and costly process and is often challenged by lack of an appropriate system, low hit rates with high false-positives and toxicity of compounds. In this study, we use a yeast cell-based HTS to identify compounds that inhibit the Aβ_42_-RF oligomerization among a library composed of FDA approved marketed drugs or drug-like compounds. Eight drug candidates emerged from the HTS screen (Figure 2), and 7 were found to have anti-Aβ_42 _oligomeric properties in subsequent biochemical assays (Figure 3): 3 antipsychotics (bromperidol, haloperidol and azaperone), 2 anesthetics (pramoxine HCl and dyclonine HCl), tamoxifen citrate, and minocycline HCl. Furthermore, the anti-oligomeric activity in growing yeast, of each of these 7 drugs, increased with increased dosages. Among our 7 hits, bromperidol, haloperidol, and azaperone are in the same drug class: dopamine antagonists. Pramoxine HCl and dyclonine HCl are both anesthetics.

Since the drugs we identified are already used in humans to treat other diseases, their side effects are known to be tolerable. In addition, at least four of these drugs - bromperidol, haloperidol, azaperone, and minocycline HCl, pass the blood brain barrier. These are both hurdles that eliminate most Alzheimer’s drug candidates from further study. Thus epidemiological studies of Alzheimer incidence in patients that take these drugs is now warranted.

Importantly, we linked the Aβ_42_-RF oligomers formed in yeast with a possible pathological toxic oligomer species of the HDEL-Aβ_42 _fusion that was directed to and disrupted normal cellular endocytic trafficking in yeast 24. While Yap1802 restored endocytic trafficking function to signaling molecules perturbed by Aβ_42_ aggregates 24, and PICALM protected rat cortical neurons from toxicity of Aβ_42_ oligomers formed extracellularly 24, it was not clear how PICALM actually impacted Aβ toxicity. Here, by testing genetic modifiers of HDEL-Aβ_42_ toxicity for those that repair the compromised Aβ_42_-RF translational termination factor activity we found that cells overexpressing YAP1802 regained translational termination factor activity, suggesting it restored Aβ_42_-RF to the soluble monomeric state (Figure 1A). Indeed, overexpressed Yap1802 reduced the level of SDS-resistant Aβ_42_-RF oligomer by more than 50% (Figure 1B), indicating that Yap1802 is likely to have a common role in aggregation and toxicity of Aβ_42_.

As expected, the other anti-Aβ_42_-RF oligomer drugs also rescued toxicity of HDEL-Aβ_42_ (Figure 5). In contrast, the other 11 modifiers of HDEL-Aβ_42 _activity (shown in Figure 1A) did not have any effects on the Aβ_42_-RF system. This is not unexpected as these modifiers may affect secretion rather than oligomer formation of the peptide. Also all of the 7 anti-Aβ_42_-RF oligomerization drugs had effects on toxicity of Aβ_42_ rat PC12 cells (Figure 4A) and another toxic yeast AD system (Figure 5). This validates the Aβ_42_-RF system as a useful screen for Alzheimer’s drugs.

In our study the relative effectiveness of each drug was not always consistent in different assays, leaving the most relevant assay to be determined. Minocycline HCl and tamoxifen citrate only suppressed Aβ_42_-RF oligomerization at high concentrations (Figure 3), but had among the strongest effects on oligomer levels and toxicity (Figures 2-5). Furthermore, only tamoxifen citrate inhibited *in vitro* oligomerization of TMR-Aβ_42_ (Figure 4D), indicating that minocycline HCl and tamoxifen citrate may inhibit Aβ_42_ toxicity via distinct pathways.

It is interesting that minocycline HCl and tamoxifen citrate, which were identified as strong hits in this study, are already being used in clinical trials for other neurodegenerative diseases, such as Amyotrophic Lateral Sclerosis and Huntington Disease (ClinicalTrials.gov ). Furthermore, minocycline HCl is a semi-synthetic tetracycline antibiotic that effectively crosses the blood-brain barrier. It has been suggested that minocycline protects patients from brain damage by reducing inflammation in AD, related tauopathies and neurodegenerative diseases that are caused by misfolded proteins 58,59,60,61. Tamoxifen is an antagonist of calmodulin, a major cellular calcium receptor and calcium dependent regulator of many cellular processes. Increased calcium level alters neuronal dysfunction and ultimately leads to cell death in AD brains, showing there is a connection between calcium and AD. We now suggest that minocycline HCl and tamoxifen citrate also have anti-Aβ_42 _oligomeric activity.

The recent detection of potential hybrid oligomers composed of Aβ and other neurodegenerative disease associated proteins such as α-synuclein, TDP-43 and PrP from human AD post mortem brains suggest that Aβ oligomers may act as a template for the aggregation of other proteins generating a secondary amyloidosis 14,15,62. Thus, prevention of Aβ peptide aggregation into toxic oligomers could not only prevent AD, but might also remove oligomeric seeds that contribute to the progression of other neurodegenerative diseases.

Our results demonstrate the efficacy of the HTS screen for drugs that inhibit Aβ_42_-RF oligomer formation. Future determination of the cellular targets of the drug hits may aid drug discovery. Most importantly it remains to be determined if these drugs can inhibit Aβ_42_ from forming toxic oligomers in humans, thereby reducing Alzheimer symptoms.

## Materials and Methods

### Yeast strains, media and plasmids

L3149 and L3150 28, used to test the effects of drugs on Aβ_42_-RF oligomerization, are *SUP35 *and *ERG6* disrupted versions of 74-D694 (*MATa ade1-14 ura3-52 his3-200 sup35*∆*::LEU2 erg6*∆*::TRP1*)*, *respectively containing p1364 (pRS313, *CEN, URA3, CUP1::Aβ_42_-RF*) or p1541 (pRS313,*CEN, URA3, CUP1::Aβ^F19,20T/I31P^-RF*). While p1364 carries the wild-type Aβ_42_ peptide fused with the M (middle) and RF (release factor) domains of Sup35 (i.e. lacking the N-terminal prion domain), called here Aβ_42_-RF, p1541 carries a F19T, F20T and I31P triple mutant Aβ_42_ peptide that is aggregation-deficient 29, called here Aβ_42_m2-RF.

To test the effects of drugs on HDEL-Aβ_42_ associated toxicity, we transformed an *ERG6* deleted strain, L3340, (*MATα ade1-14 ura3-52 leu2-3, 112 his3-200 erg6*∆*::TRP1*) with a 2µ gateway expression vector (pAG425 base, *LEU2*) carrying Aβ_42_ fused to the C-terminus of an ER retention signal (HDEL) expressed with the inducible *GAL1 *promoter, called here HDEL-Aβ_42_. The plasmid was generated by transferring *HDEL-Aβ_42_* from the pAG305 *GAL1:*:*HDEL-Aβ_42_* integrative vector 24 to pAG425GAL-*ccdB *(Addgene) using Gateway Technology 63.

The plasmids of 12 genes previously shown to suppress HDEL-Aβ_42_ toxicity 24 were kindly supplied by Susan Lindquist (Whitehead Institute for Biomedical Research, Massachusetts Institute of Technology, Cambridge, MA). Each plasmid carries the yeast ORF under the control of the *GAL1* promoter on the single copy plasmid, pBY011 (*CEN, URA3, Amp^R^*) 24,63.

Media, cultivation and transformation procedures were standard 64. Expression of Aβ_42_-RF and HDEL-Aβ_42_ were respectively driven by the copper-inducible *CUP1* promoter with 50 μM CuSO_4 _and the galactose inducible *GAL1* promoter with 2% galactose.

### Growth assays for the primary screen and confirmation

The primary screen employed the Prestwick Chemical Library® of 1,200 FDA approved or drug-like small compounds (Prestwick Chemical). Assays for selection of compounds that reduce the level of Aβ_42_-RF oligomers were as described previously 28. Drugs in DMSO were diluted to 20 µM in assay media (2% dextrose without or with adenine + 50 µM CuSO_4_, respectively, for the -Ade or +Ade assay) inoculated with 1 x 10^5^ or 1 x 10^4^ cells/well, respectively, for the -Ade or +Ade assays. Each 384-well plate contained 32 positive (Aβ_42_m2-RF) and 32 negative (Aβ_42_-RF) controls with 0.2 μl of DMSO. The OD_600 _was measured after 5 days for -Ade and 4 days for +Ade plates incubated at room temperature with shaking (900 rpm). Each assay was performed in duplicate in clear flat-bottom 384-well plates (ScreenMates) using a Tecan Freedom EVO 200 liquid handling robot at the Research Resources Center at the University of Illinois, Chicago. Drug candidates that caused less than 50% and more than 70% growth in -Ade and +Ade, respectively, compared to growth in DMSO controls were selected for further analysis. Drug effects on Aβ_42_-RF oligomer formation were directly tested with SDS gel electrophoresis using 10% Mini-PROTEAN® TGX™ precast gels (Bio-Rad) and immunoblot analysis as described previously 65. To detect the amount of SDS-resistant small oligomers, lysates were treated with 1% SDS for 7 min at room temperature, subjected to immunoblot analysis, and probed with antibodies against Sup35’s RF domain (BE4, developed by Viravan Prapapanich, a former post doc in our laboratory).

### Aβ_42_ *in vitro* oligomerization and fibrilization 

Aβ_42_ was polymerized using conditions favorable for the formation of Aβ_42_ small soluble oligomers 38 or fibrils 42. For Aβ_42_ soluble oligomer formation, synthetic recombinant peptide (HFIP, Rpeptide) dissolved to 5 mM in anhydrous DMSO (Sigma) and sonicated was diluted to 200 µM in PBS (20 mM NaH_2_PO_4_, 140 mM NaCl, pH 7.4) supplemented with 0.2% SDS and allowed to oligomerize for 24 hrs at 37°C without agitation, in the absence (DMSO control), or presence of 100 µM drug. Finally, any high molecular Aβ_42 _aggregates formed were removed by a brief centrifugation at 2,000 rpm. For Aβ_42 _fibril formation, 50 µM of each drug was added to 20 µM Aβ_42_ peptide in Tris-buffered saline (25 mM Tris, 150 mM NaCl, pH 7.5) and incubated under Aβ_42_ fibrilization conditions 42. Following incubation at 37°C for different lengths of time, 100 µl of Aβ_42_ was aliquoted into a 96 well plate with 200 µl of Thioflavine T (ThT) solution (7 µl ThT, 50 µM glycine, pH 7.1). Quantification of fibril formation was measured at 483 nm with excitation at 450 nm on a Spectra Max M5 plate reader (Molecular Devices).

### PC12 cell toxicity

Treatment of rat PC12 neuronal cells with Aβ_42_ aggregates formed under the oligomerization condition described above in the presence of each drug and subsequent assays for cell viability were as described 41. Briefly, PC12 cells, derived from rat adrenal medulla pheochromocytoma (American Type Culture Collection), were cultured in RPMI 1640 complete growth medium (ATCC) supplemented with horse serum and fetal bovine serum and grown to confluency at 37°C with 5% CO_2_. Cells were plated in tissue culture-treated flat bottom 96-well plates (Corning) at 10,000 cells/well and allowed to attach overnight before adding Aβ_42_ aggregates. Following incubation of cells for 24 hrs, the MTT cell viability assay was used to determine cell toxicity (Roche Applied Science). Briefly, 10 µl of MTT was added to each well and following incubation for 4 hrs at 37°C, 100 µl of solubilization solution was added and incubated overnight at 37°C. Absorbance was then recorded at 570 nm.

### Oligomerization of TMR-labeled Aβ_42 _

As described previously 43 a stock solution of TMR-K-Aβ_42_ (42 µM) in 4 M GdnCl was diluted to a final concentration of 1 µM in PBS (pH 7.4) supplemented with 1 mM EDTA and 5 mM β-mercaptoethanol. Drugs (10 µM) were added to the reaction. The fluorescence of TMR-K-Aβ_42_ was recorded as a function of time immediately after dilution of the TMR-Aβ_42_ in an Alphascan fluorometer (Photon Technology International), with excitation and emission monochromators set to 520 and 600 nm, respectively.

## SUPPLEMENTAL MATERIAL

Click here for supplemental data file.

All supplemental data for this article are also available online at http://microbialcell.com/researcharticles/inhibition-of-a%CE%B242-oligomerization-in-yeast-by-a-picalm-ortholog-and-certain-fda-approved-drugs/.
