# Supplementary material for: Inhibition of Aβ42 oligomerization in yeast by a PICALM ortholog and certain FDA approved drugs
Source: Microb Cell. 2016 Jan 20;3(2):53–64. doi: 10.15698/mic2016.02.476 (PMC5349104; doi:10.15698/mic2016.02.476)
Supplement: Supplementary file 1 [file mic-03-053-s01.pdf]

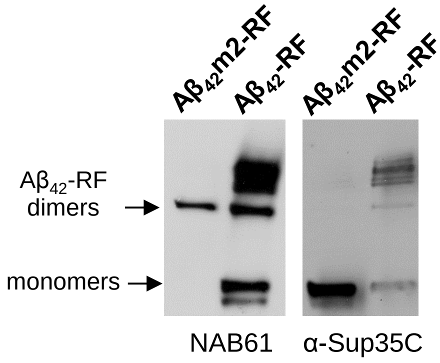

Figure S1. Detection of toxic Aβ$_{42}$-RF oligomers using NAB61, an Aβ oligomer-selective monoclonal antibody. The prediction of the Aβ$_{42}$-RF migration positions were based on the migration of the Aβ$_{42}$-RF monomer (calculated molecular mass 73.7 kDa), which migrated at 95 kDa on PAGE.

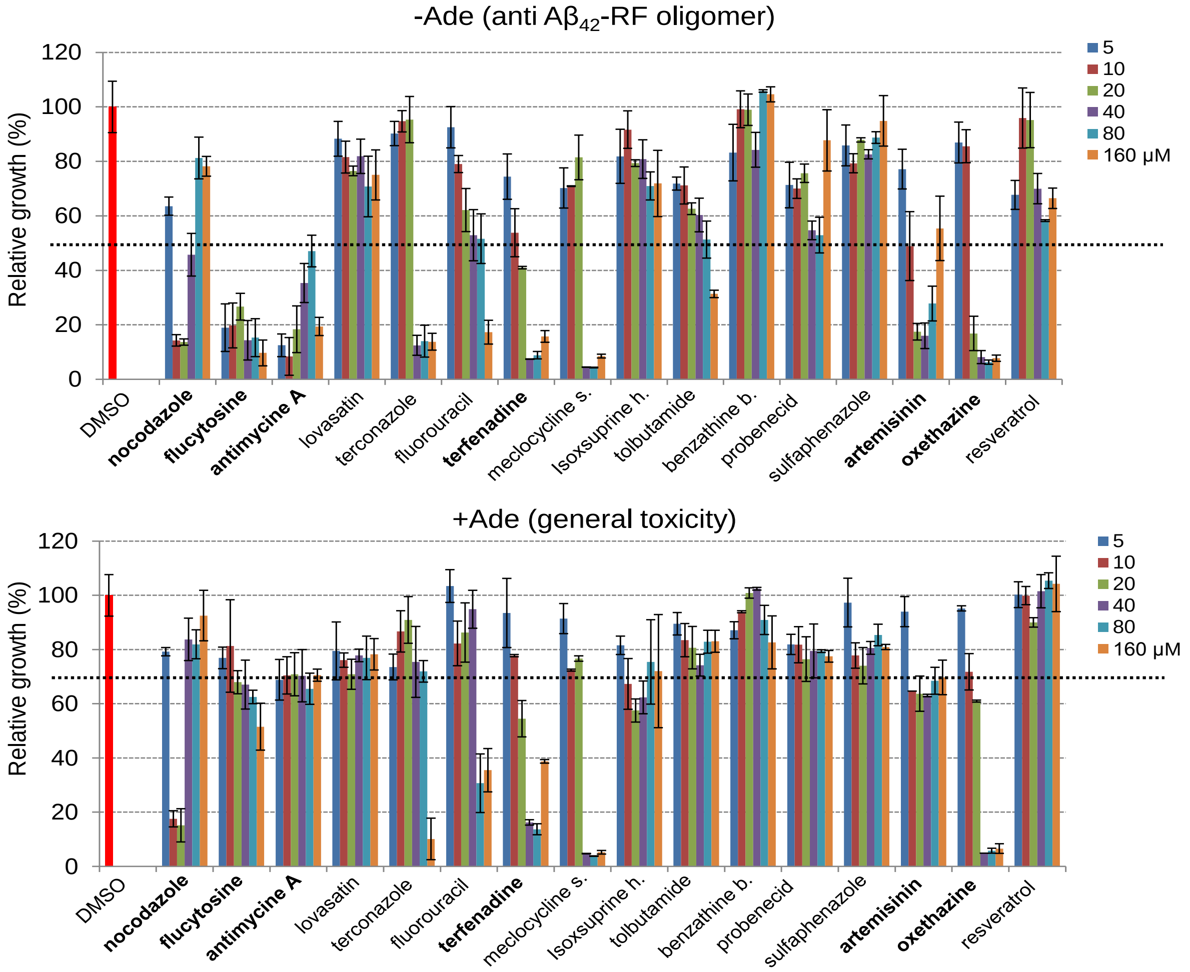

Figure S2. The effect of 16 drugs on Aβ_{42}-RF translational misreading and general cell growth. To exclude false positive drugs the effect of each drug on Aβ_{42}-RF translational termination factor activity and general growth was measured. The experimental protocol is as in Figure 1. Drugs in bold at 20 µM that exhibited more than 50% growth (dotted line) in –Ade or less than 70% growth (dotted line) in +Ade, were dropped due to inactivity or a general toxic effect (nocodazole, flucytosine, terfenadine oxethazine). The data shows the average of three replicates. Error bars represent the standard deviation.

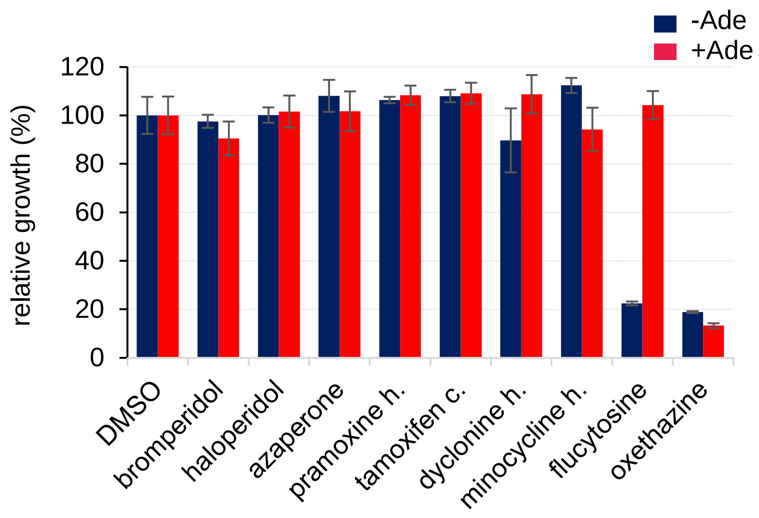

Figure S3. The effects of drugs on the ability of a *sup35* suppressor mutant to read-through the *ade1-14* nonsense mutation. To eliminate the possibility that general antisuppression, which impairs translational termination activity, rather than specific effects on $A\beta_{42}$-RF, caused the *ade1-14* premature stop codon to be read-through, each drug was tested in L3345 carrying a *SUP35 (G1256A* [37]*)* suppressor mutation in a 74-D694 derivative (*MATa ade1-14 ura3-52 leu2-3,112 trp1-289 his3-200, erg6∆::TRP1*). For the –Ade or +Ade assays, respectively, $1{\times}10^5$ or $1{\times}10^4$ cells/well were inoculated in the presence of 44 µM of each drug. OD_{600} was measured after 3 days. The 7 drug candidates had no significant growth change in –Ade vs. +Ade compared to the DMSO control. In contrast flucytosine is a general antisuppressor that reduced read-through, and thus reduced growth on -Ade. Oxethazine is a general toxic control. Shown is the relative growth in the presence vs. absence of each drug (DMSO). Error bars show the standard deviation from three replicates.

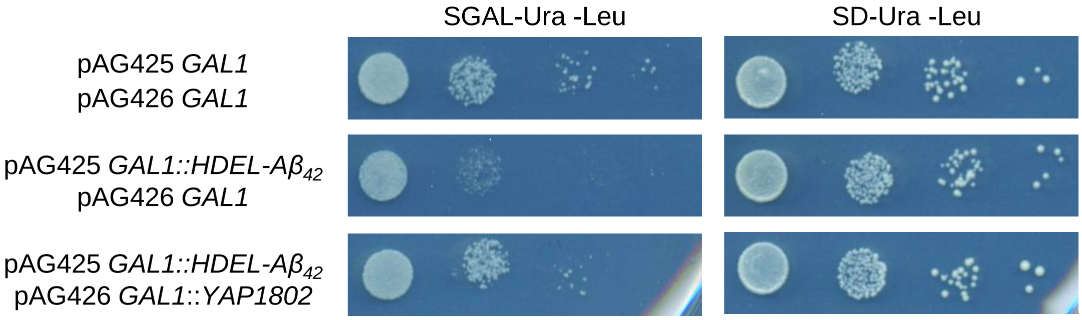

Figure S4. *YAP1802* overexpression suppressed the toxicity of HDEL-Aβ_{42} overexpression in *ERG6* disrupted yeast. L3340 (*MATα ade1-14 ura3-52 leu2-3, 112 his3-200 erg6Δ::TRP1*) was cotransformed with a 2µ gateway expression vector carrying Aβ_{42} fused to an ER retention signal (*HDEL*) under control of *GAL1* promoter (pAG425 *GAL1::HDEL*-Aβ_{42}, *LEU2*) and an empty vector (pAG426 *GAL1, URA3*) or *YAP1802* (pAG426 *GAL1::YAP1802*). Tenfold dilutions of exponentially growing cultures were spotted onto SD-Ura –Leu and SGAL-Ura –Leu (↑HDEL-Aβ_{42} ↑Yap1802) and incubated at 30°C for 4 days.

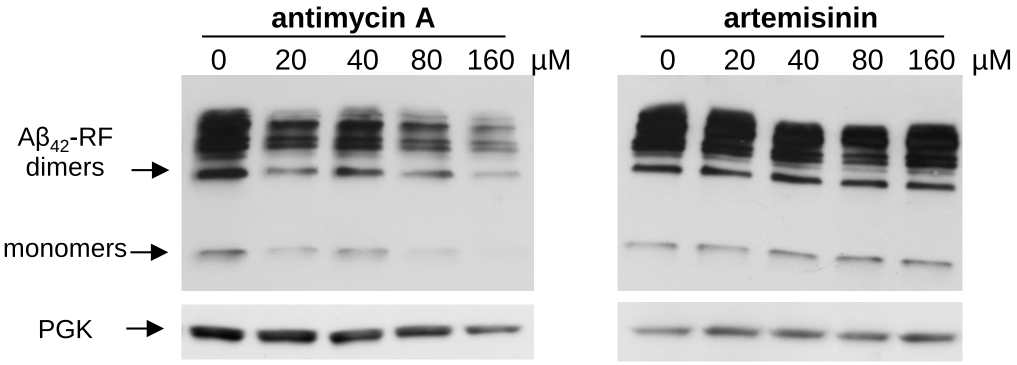

Figure S5. Two false positive drugs have no effect on the level of Aβ_{42}-RF oligomerization. Immunoblots of lysates prepared from the assay strain expressing Aβ_{42}-RF, grown in the presence of antimycine A or artemisinin at the indicated concentrations, were developed with anti-Sup35 RF antibodies. PGK, detected with anti-PGK antibodies, was used as an internal control.
